# ﻿Two new species of *Gymnopus* sect. *Levipedes* (Omphalotaceae, Agaricales) from Central and North China

**DOI:** 10.3897/mycokeys.118.143277

**Published:** 2025-05-22

**Authors:** Jia-Jun Hu, Yong-Lan Tuo, Zheng-Xiang Qi, Dong-Hua Jiang, Yu Li, Bo Zhang

**Affiliations:** 1 College of Life Science, Zhejiang Normal University, Jinhua City, Zhejiang Province, 321004, China; 2 Joint Laboratory of International Cooperation in Modern Agricultural Technology, Ministry of Education, Jilin Agricultural University, Changchun City, Jilin Province, 130118, China; 3 Engineering Research Center of Edible and Medicinal Fungi, Ministry of Education, Jilin Agricultural University, Changchun City, Jilin Province, 130118, China

**Keywords:** Cheilocystidia, ecology, *Gymnopusdryophilus* complex

## Abstract

The genus *Gymnopus* has a long research history and is known for its high species diversity worldwide. However, its species diversity in China remains poorly understood. Through a combination of detailed morphological studies and phylogenetic analysis, this study described two new species from Northeast and Central China, *Gymnopusbiyangensis* and *Gymnopussinodryophilus*, both belonging to Gymnopussect.Levipedes. *Gymnopusbiyangensis* is characterized by basidiomata appearing in summer and originating from broad-leaved forests, a dark reddish-brown pileus, a cylindrical to clavate stipe, and clavate to cylindrical, diverticulate cheilocystidia. *Gymnopussinodryophilus* differs by the yellowish-white to light brown basidiomata arising from coniferous and broad-leaved mixed forests, coralloid but inflated pileipellis terminal cells, and an apex of cheilocystidia not coralloid. Additionally, a key to the reported species of Gymnopussect.Levipedes in China is provided.

## ﻿Introduction

*Gymnopus* (Pers.) Gray has a long research history dating back to 1801 (Persoon 1801) and plays a significant role in various ecosystems (Mata et al. 2006). In many countries, including China and elsewhere, certain species of *Gymnopus* are also treated as food resources (Hu and Zhang 2023). As with many macrofungi, *Gymnopus* (previously classified under the genus *Collybia*) was first studied in Europe, with significant contributions made by European mycologists (Persoon 1801; Fries 1821; Antonín et al. 1997; Antonín and Noordeloos 1997). Over time, this research enthusiasm spread to other regions (Cooper and Leonard 2013; Antonín et al. 2014; Coimbra et al. 2015; Deng 2016; Oliveira et al. 2019; Hu et al. 2022a, b), particularly North America, where many studies have been conducted (Halling 1996; Desjardin et al. 1999; Mata et al. 2006; Petersen and Hughes 2014).

However, compared to other genera, such as *Agaricus* L. and *Cantharellus* Adans. ex Fr., *Gymnopus* has not been as thoroughly studied. This discrepancy in research interest is partly due to regional differences in mycologists’ areas of focus, which, have led to significant research gaps between *Gymnopus* and more widely studied genera. For example, approximately 300 species of *Gymnopus* have been described globally (Hu et al. 2022a, b), with the majority of species documented in North America, Europe, and Asia (Sun et al. 2021b; Hu and Zhang 2023). In contrast, fewer species have been reported in Oceania and Africa (Hu and Zhang 2023).

In recent decades, Asia has emerged as a new hotspot for *Gymnopus* research, evidenced by the discovery of new species or new combinations (Hu and Zhang 2023). This indicates an increasing recognition of the genus’ biodiversity and ecological importance. Nevertheless, the full extent of *Gymnopus* species diversity remains underappreciated and warrants further exploration.

SubsectionLevipedes is characterized by a stipe that is smooth, polished, or pubescent; a pileipellis that typically forms an entangled (never radially oriented) trichoderm, consisting of inflated, often lobed elements or coralloid “*dryophila*-type” structures; trama and its elements are non-dextrinoid, and usually saprophytic, commonly found in coarse humus, forest litter, or on decaying wood (Halling 1996; Antonín and Noordeloos 2010; Oliveira et al. 2019). In this subsection, some species belonging to the *Gymnopusdryophilus* (Bull.) Murrill complex have historically been treated as food resources in Northeast and Southwest China (Hu and Zhang 2023). Despite this, recent studies have shown that the species complex, *Gymnopusdryophiloides*, can cause diarrhea in humans (Ma et al. 2024). Furthermore, due to the similarity in appearance, difficulties remain in identifying the species of this complex. Although Vilgalys and Miller (1983, 1987a, b) and Antonín et al. (2013) have carefully studied the North American or European *G.dryophilus* species complex and identified key characteristic features, all these results were based on the North American or European specimens. Additionally, there is still a lack of comprehensive study on the complex outside of North America and Europe.

Looking back at the research history, it is evident that taxonomic studies are mainly focused on Europe and North America, leaving a significant gap on other continents. As research on *Gymnopus* deepens, taxonomic issues and questions about the classification of the genus emerge, highlighting the need for further attention to this group. Consequently, taxonomic research on *Gymnopus* has begun in China. Specimens of the *G.dryophilus* complex collected from Henan and Jilin Provinces are studied in detail here. As a result, two new species belonging to Gymnopussect.Levipedes are described and illustrated. This study enriches our understanding of the species diversity of *Gymnopus* and provides valuable insights for future studies.

## ﻿Materials and methods

### ﻿Specimen collection

The specimens used in this study were collected between 2020 and 2023 in Henan and Jilin Provinces, China. All specimens were photographed in situ, with an emphasis on capturing basidiomata at various stages of development. Subsequently, three or more basidiomata were collected for detailed morphological and molecular analysis. The morphological characteristics, including size, color, and odor, were documented. The color references followed Flora of British Fungi: Color Identification Chart (Royal Botanic Garden 1969). A clean tissue sample from each specimen was dried using allochroic silica gel for DNA extraction. The specimens were dried in an electric oven at approximately 45 °C.

### ﻿Identification

The recognition and description of macro-characteristics were based on field notes and photographs. Dried specimens were rehydrated in 94% ethanol and subsequently mounted in 3% potassium hydroxide (KOH), 1% Congo Red, or Melzer’s Reagent for examination. Structures such as basidiospores, basidia, and cheilocystidia were observed using a Zeiss Axio Lab. A1 microscope. For each specimen, a minimum of 40 measurements were taken from at least two different basidiomata. The size of basidiospores is expressed as length × width (L × W). To account for size variation, 5% of the measurements from each end of the range were excluded, and the final measurements are given as (a) b × c (d). Q represents the ratio of L to W for each studied specimen, while Qm denotes the average Q value ± standard deviation. The examined specimens are deposited in the Herbarium of Mycology at Zhejiang Normal University (ZNU-F).

### ﻿DNA extraction, PCR, and sequencing

Total DNA was extracted from dried materials using the NuClean Plant Genomic DNA Kit (Kangwei Century Biotechnology Company Limited, Beijing, China), following the manufacturer’s instructions. The internal transcribed spacer (ITS) region, nuclear large ribosomal subunits (nLSU), and translation elongation factor (*tef-1α*) loci were selected for phylogenetic analysis. The primer pairs ITS4-ITS5 (Gardes and Bruns 1993), LROR-LR5/LR7 (Vilgalys and Hester 1990; Cubeta et al. 1991), and 983F-1567R (Rehner and Buckley 2005) were used to amplify the ITS, nLSU, and *tef-1α*, respectively.

PCR reactions (25 μL) were prepared as follows: 8.5 μL of dd H_2_O, 12.5 μL of 2 × Taq PCR MasterMix, 1 μL of each primer, and 2 μL of DNA sample. The reaction conditions were based on those described by Coimbra et al. (2015) for ITS, Ryoo et al. (2020) for nLSU, and Xu et al. (2021) for *tef1-α*. PCR products were visualized under UV light following electrophoresis on 1% agarose gels stained with ethidium bromide. The PCR products were then sent to Hangzhou Huada-Qinglan Innovation Technology Co., Ltd. for sequencing, using the Sanger method. The new sequences were deposited in GenBank (http://www.ncbi.nlm.nih.gov/genbank), and the detailed sequence information is provided in Table 1.

**Table 1.** Voucher/specimen numbers, country, and GenBank accession numbers of the specimens included in this study. Sequences produced in this study are in bold and obtained from type materials marked as T.

Scientific name	Country	Specimen/Voucher numbers	GenBank Accession Numbers		
			ITS	nLSU	*tef1-α*
*Collybiopsisjuniperinus*	USA	TENN59540	AY256708	KY019637	–
*Collybiopsisobscuroides*	Norway	GB-0150514	KX958399	KX958399	–
*Collybiopsissubnuda*	USA	TENN-F-61138	KY026667	FJ750262	–
*Gymnopusabruptibulbus* nom. prov.	China	HMJAU61050	OQ597084	–	–
*Gymnopusalkalivirens*	USA	TENN51249	DQ450000	–	–
*Gymnopusalliifoetidissimus*	China	GDGM76695	MT023344	MT017526	–
*Gymnopusalpicola*	Spain	BRNM705055	MK278102	MK278102	–
*Gymnopusalpinus*	Latvia	CB16251	JX536168	–	JX536191
*Gymnopusandrosaceus*	Russia	TENN-F-59594	KY026663	KY026663	–
*Gymnopusandrosaceus*	France	CBS239.53	MH857174	MH868713	–
*Gymnopusaquosus*	Czech Republic	BRNM665362	JX536172	–	JX536192
*Gymnopusatlanticus* (T)	Brazi	URM87728	KT222654	KY302698	–
*Gymnopusaurantiipes*	–	AWW118	AY263432	AY639410	–
*Gymnopusaustrosemihirtipes* (T)	Indonesia	SFSU-AWW65	AY263422	–	–
*Gymnopusbarbipes*	USA	TENN67855	KJ416269	NG_059733	–
*Gymnopusbicolor*	–	AWW116	AY263423	AY639411	–
*Gymnopusbisporus*	Spain	BCN-SCM B-4065	JN247551	JN247555	–
***Gymnopusbiyangensis*** (T)	**China**	**ZNU-F-001**	**PQ651934**	**PQ651940**	**PQ661922**
***Gymnopusbiyangensis***	**China**	**ZNU-F-002**	**PQ651935**	**PQ651941**	**PQ661923**
***Gymnopusbiyangensis***	**China**	**ZNU-F-003**	**PQ651936**	**PQ651942**	**PQ661924**
*Gymnopusbrassicolens*	Russia	TENN55550	DQ449989	–	–
*Gymnopusbrunneiniger* (T)	Mexico	XAL-Cesar 49	MT232389	NG-075396	–
*Gymnopusbrunneodiscus*	Korea	BRNM 808975	MH589975	MH589991	–
*Gymnopuscampanifomipileatus* nom. prov.	China	HMJAU61027	OQ597064	OQ594474	–
*Gymnopuscatalonicus*	Spain	BCN-SCM B-4057	JN247552	JN247556	–
*Gymnopusceraceicola* (T)	New Zealand	PDD87181	KC248405	–	–
*Gymnopuschangbaiensis* (T)	China	HMJAU60300	OM030272	OM033387	–
*Gymnopuscremeostipitatus* (T)	Korea	BRNM747547	KF251071	KF251091	–
*Gymnopuscystidiosus* (T)	China	HMJAU60992	ON259024	ON259036	–
*Gymnopusdensilamellatus* (T)	Korea	BRNM714927	KP336685	KP336694	–
*Gymnopusdryophilus*	Czech Republic	BRNM695586	JX536143	–	JX536196
*Gymnopusdryophilus*	Japan	Duke31	DQ480099	–	–
*Gymnopusdryophioides* (T)	South Korea	BRNM781447	MH589967	MH589985	–
*Gymnopusdysodes*	USA	TENN-F-61125	KY026666	KY026666	–
*Gymnopusearleae*	USA	TENN-F-59140	DQ449994	KY019634	–
*Gymnopusefibulatus* (T)	China	HGASMF01-7052	OM970865	OM970865	–
*Gymnopusepiphyllus* (T)	China	HMJAU60990	ON259030	ON259038	–
*Gymnopuserythropus*	Czech Republic	BRNM714784	JX536136	–	JX536183
*Gymnopusfagiphilus*	Czech Republic	BRNM707079	JX536129	–	JX536209
*Gymnopusfoetidus*	USA	TENN-F-65806	KY026682	KY026682	–
*Gymnopusfuscopurpureus*	Spain	BRNM-809119	MZ542559	MZ542563	–
*Gymnopusfusipes*	Austria	TENN59300	AF505777	–	–
*Gymnopusfusipes*	France	TENN59217	AY256710	AY256710	–
*Gymnopusglobulosus* (T)	China	HMJAU60307	OM030269	OM033406	–
*Gymnopusgraveolens*	France	FF17084	MH422573	MH422572	–
*Gymnopushakaroa* (T)	New Zealand	PDD87315	KC248410	–	–
*Gymnopushemisphaericus* nom. prov.	China	HMJAU61077	OQ597057	OQ594467	–
*Gymnopushybridus*	Italy	BRNM695773	JX536177	–	JX536208
*Gymnopusimbricatus* (T)	New Zealand	PDD95489	KC248390	–	–
*Gymnopusimpudicus*	Russia	TENN60094	KJ416263	–	–
*Gymnopusindoctoides*	Singapore	AY263424	AY639419	–	–
*Gymnopusinexpectatus*	Italy	–	EU622905	EU622906	–
*Gymnopusinusitatus*	Spain	BCN-SCM B-4058	JN247553	JN247557	–
Gymnopusinusitatusvar.cystidiatus (T)	Hungary	BRNM737257	JN247550	JN247554	JX536179
*Gymnopusiocephalus*	USA	TENN52970	DQ449984	KY019630	–
*Gymnopusiodes* (T)	China	HGASMF01-10068	OM970869	OM970869	–
*Gymnopusirresolutus*	Sao Tome	SFSU-DED-8209	MF100973	–	–
*Gymnopusjunquilleus* (T)	USA	TENN55224	NR_119582	–	–
*Gymnopuskauffmanii*	USA	DUKE230	DQ450001	–	–
*Gymnopuslachnophyllus*	USA	NAMA2015-320	MH910564	–	–
*Gymnopuslanipes* (T)	Spain	BRNM670686	JX536137	–	JX536205
*Gymnopusloiseleurietorum*	Sweden	URM 90060	KY321571	KY321572	–
*Gymnopuslongisterigmaticus* (T)	China	HMJAU60288	OM030282	OM033403	–
*Gymnopuslongistipes* (T)	China	HMJAU61076	PP646156	PP646168	PP654450
*Gymnopuslongus* (T)	China	HMJAU60291	OM030285	OM033400	–
*Gymnopusmacropus*	Costa Rica	TENN58619	DQ449979	–	–
*Gymnopusmacrosporus* (T)	China	HMJAU60294	OM030266	OM033397	–
*Gymnopusmontagnei*	Brazil	URM87715	KT222652	–	–
*Gymnopusneobrevipes* (T)	USA	TENN-F-14505H1	MH673477	MH673477	–
*Gymnopusnubicola*	Costa Rica	NYBG REH 8290	AF505781	–	–
*Gymnopusocior*	Czech Republic	BRNM699795	JX536166	–	JX536188
*Gymnopusomphalinoides* (T)	China	GDGM 78318	MW134044	MW134730	–
*Gymnopuspallipes* (T)	China	GDGM81513	MW582856	–	–
*Gymnopuspolyphyllus*	USA	TENN59455	AY256695	–	–
*Gymnopuspubipes*	Spain	AH26931	MZ542558	MZ542562	–
*Gymnopuspygmaeus*	Brazil	URM90003	KX869966	KY088273	–
*Gymnopussalakensis*	Indonesia	SFSU-AWW29	AY263447	–	–
*Gymnopusschizophyllus* (T)	China	GDGM 77165	MW134043	MW134729	–
*Gymnopussemihirtipes*	USA	TENN-F-07595	OK376741	–	–
*Gymnopussepiiconicus*	Indonesia	SFSU-AWW126	AY263449	–	–
*Gymnopussimilis* (T)	Korea	BRNM766739	KP336692	KP336699	–
***Gymnopussinodryophilus***	**China**	**ZNU-F-004**	**PQ651937**	**PQ651943**	**PQ661925**
***Gymnopussinodryophilus*** (T)	**China**	**ZNU-F-005**	**PQ651938**	**PQ651944**	**PQ661926**
***Gymnopussinodryophilus***	**China**	**ZNU-F-006**	**PQ651939**	**PQ651945**	**PQ661927**
*Gymnopusspadiceus* (T)	China	HMJAU61205	PP646160	PP646172	PP654454
*Gymnopusspongiosus*	USA	TENN-F-68184	KY026706	KY026706	–
*Gymnopusstriatipileatus* (T)	China	HMJAU61073	PP646166	PP646178	–
*Gymnopusstriatus* (T)	China	HMJAU60297	OM030263	OM033384	–
*Gymnopusstrigosipes* (T)	China	HMAS295796	OM970874	OM970874	–
*Gymnopussubdensilamellatus* (T)	China	HMJAU60997	ON259032	ON259042	–
*Gymnopussubpolyphyllus* (T)	China	HMJAU60999	ON259028	ON259043	–
*Gymnopussubsulphureus*	USA	TENN56321	DQ449972	–	–
*Gymnopussubsupinus*	New Zealand	PDD96595	KM975399	KM975375	–
*Gymnopustalisiae* (T)	Brazil	URM87730	KT222655	KX958401	–
*Gymnopustiliicola* (T)	China	HMJAU60305	OM030275	OM033393	–
*Gymnopustomentosus* (T)	China	HMJAU60303	OM030278	OM033390	–
*Gymnopustrabzonensis* (T)	Turkey	KATO Fungi 3375	KT271754	–	–
*Gymnopusvariicolor* (T)	Korea	BRNM714959	LT594121	KP348011	–
*Gymnopusviridiscus* (T)	China	HMJAU61202	PP646159	PP646171	PP654453
*Gymnopusvitellinipes* (T)	Indonesia	SFSU-AWW127	AY263429	AY639432	–
*Marasmiusaurantioferrugineus*	South Korea	BRNM714752	FJ904962	MK278334	–
*Marasmiusbrunneospermus* (T)	South Korea	KPM-NC0005011	FJ904969	FJ904951	–

### ﻿Phylogenetic analysis

Based on the BLASTn results and morphological similarities, sequences related to these samples were collected (Table 1). A combined dataset of ITS, nLSU, and *tef-1α* fragments, consisting of 85 sequences obtained from the type species of *Gymnopus* and *Marasmius* Fr., was used for phylogenetic analysis. Species belonging to *Marasmius*, *Marasmiusaurantioferrugineus* Hongo and *Marasmiusbrunneospermus* Har. Takah., were selected as outgroups (Hu et al. 2024).

Each gene region in the dataset was aligned using MAFFT 7.490 (Katoh and Standley 2013) and subsequently manually inspected in BioEdit 7.0.5.3 (Hall 1999). The alignments of the ITS, nLSU, and *tef-1α* sequences were then combined through PhyloSuite 1.2.2 (Zhang et al. 2020). A partition homogeneity test (PHT) (Farris et al. 1994) was performed on the multi-gene dataset with PAUP 4.0b10 (Swofford 2002), employing 1000 homogeneity replicates. The best-fit evolutionary model was estimated using ModelFinder (Kalyaanamoorthy et al. 2017). Bayesian inference (BI) was applied for phylogenetic analysis, utilizing MrBayes 3.2.6 with a general time-reversible DNA substitution model and gamma distribution rate variation across the sites (Ronquist and Huelsenbeck 2003). Four Markov chains were run for two independent runs, starting from random trees, until the split frequency value fell below 0.01. Trees were sampled every 100 generations, with the first 25% discarded as burn-in. The remaining trees were used to construct a 50% majority consensus tree and calculate the Bayesian posterior probabilities (PP). Maximum likelihood (ML) analysis was performed using RaxmlGUI 2.0.10 (Edler et al. 2021) with 1000 bootstrap (BS) replicates to search for the optimal topology. The resulting trees were visualized using FigTree 1.4.4 (http://tree.bio.ed.ac.uk/software/figtree/, accessed 25 October 2020).

## ﻿Results

### ﻿Phylogenetic analysis

**Figure 1.**
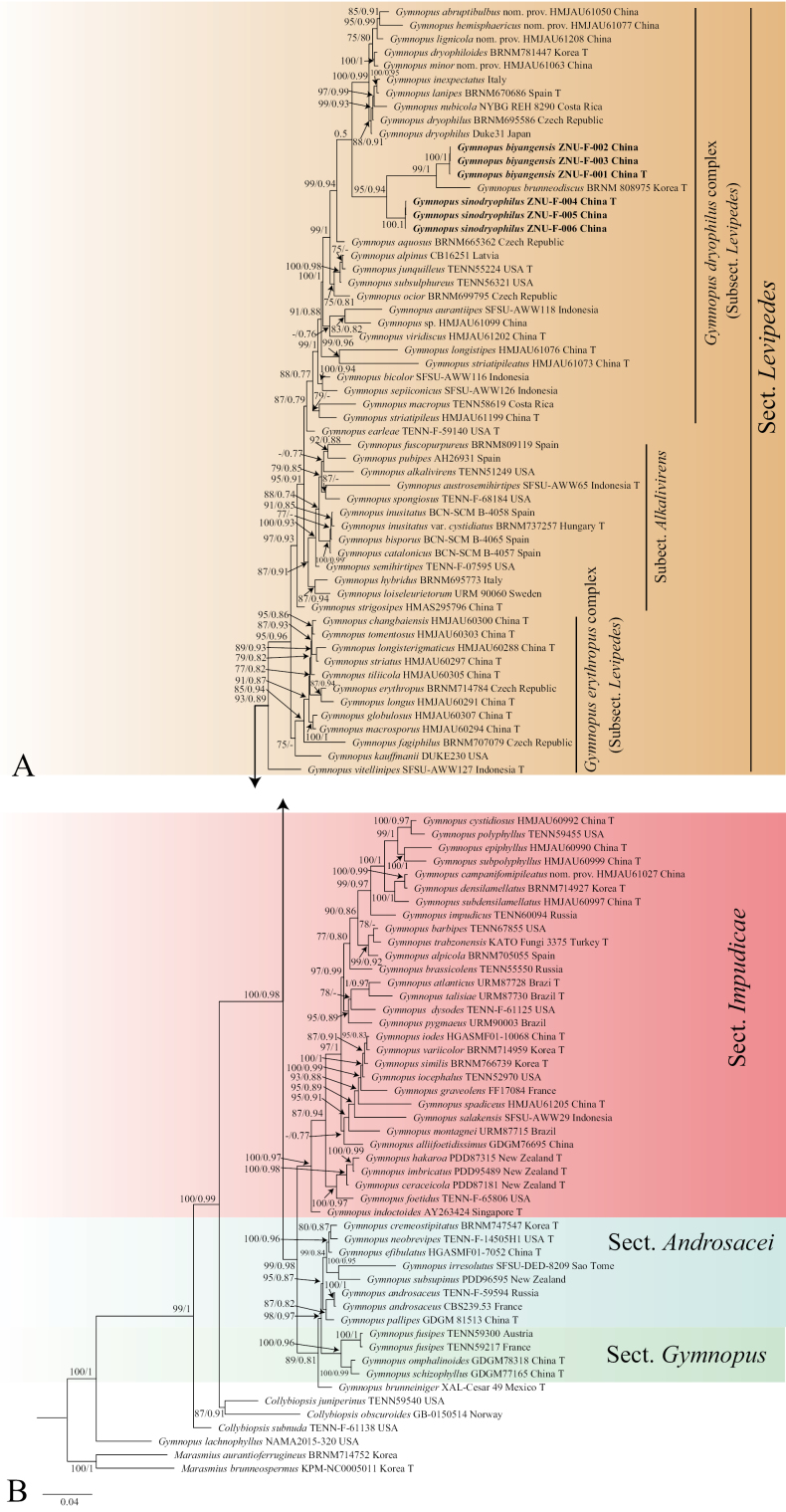
Maximum likelihood analysis generated from the combined ITS, nLSU, and *tef-1α* dataset of genus *Gymnopus*. Bootstrap values (BS) ≥ 75% from ML analysis and Bayesian posterior probabilities (PP) ≥ 0.80 are shown on the branches. Newly sequenced collections are indicated in bold, and the type specimens are denoted by (T).

A total of 18 new sequences (six per locus) were obtained from six samples in this study. In the combined dataset, 189 sequences derived from three gene loci (ITS, nLSU, and *tef-1α*) from 102 samples were used for phylogenetic analysis. The best-fitting model for BI was GTR+F+I+G4, while the GTRGAMMA model was applied for ML (Vizzini et al. 2015). The Bayesian analysis was run for four million generations, resulting in an average standard deviation of split frequencies of 0.004528. The same dataset and alignment were also analyzed using the ML method. Both phylogenetic analyses yielded a similar topology, which is depicted in Fig. 1.

Our phylogenetic analysis revealed that species of the *G.dryophilus* complex form a distinct clade, which is sister to the *Gymnopuserythropus* (Pers.) Antonín, Halling & Noordel. complex. Two newly proposed species are independently positioned within the genus *Gymnopus*, with strong phylogenetic evidence.

### ﻿Taxonomy

#### ﻿ Gymnopus biyangensis

Taxon classificationFungiAgaricalesOmphalotaceae

J.J. Hu, B. Zhang, X. Li & Y. Li sp. nov.

87617FF1-925E-5083-8D1A-F43F68E49494

Fungal Names: FN 572234

Figs 2A , 3

##### Etymology.

Refers to the location of type material.

##### Diagnosis.

[English] This species is characterized by the basidiomata that appear in summer and originate from broad-leaved forests, dark reddish-brown pileus, cylindrical to clavate stipe, clavate to cylindrical cheilocystidia with a narrowly protruding apex.

**Figure 2.**
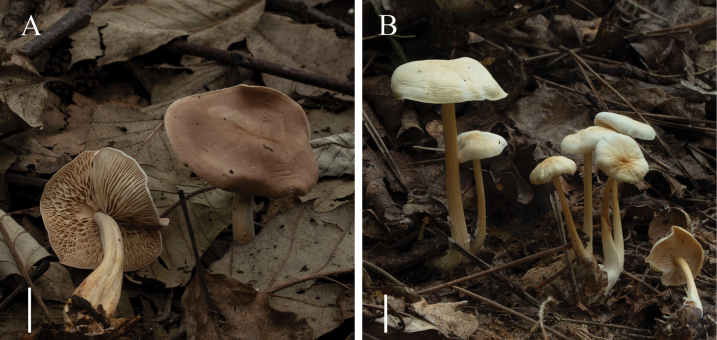
The habit of *Gymnopus* spp. described in this study: **A***Gymnopusbiyangensis***B***Gymnopuschangchunensis*. Scale bars: 1 cm.

##### Type.

China. Henan Province • Zhumadian City, Biyang County, Mingzhuang Village, 10 July 2021, Jia-Jun Hu, Bo Zhang, and Xiao Li, ZNU-F-001 (Collection No.: Hu 769), holotype.

**Figure 3.**
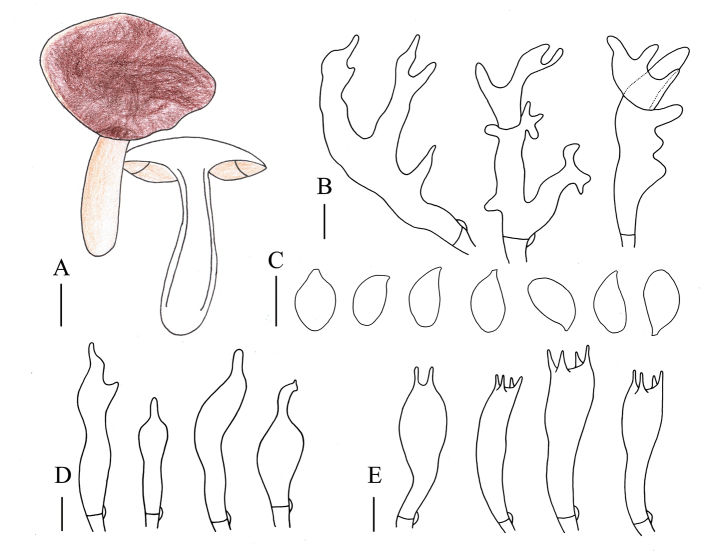
Morphological characteristics of *Gymnopusbiyangensis* (ZNU-F-001) **A** basidiomata **B** pileipellis elements **C** basidiospores **D** cheilocystidia **E** basidia. Scale bars: 1 cm (**A**); 5 µm (**B–E**).

##### Description.

Basidiomata medium-sized. Pileus 3.3–4.4 cm in diameter, applanate-convex, reddish brown to brown, smooth, glabrous; margin entire, wavy to upturned, dark reddish-brown to light brown. Context thin, yellow to light brown, freshy, odorless. Stipe 2.0–5.0 cm long and 0.5–1.3 cm wide, central, cylindrical to clavate, smooth, light yellow, occasionally with reddish brown tones or slight spots at the base. Lamellae adnate to adnexed, close, yellowish brown to light brown, unequal. Occurrence in leaf litter.

Basidiospores 5.0–6.0 × 3.0–4.0 µm, Q = (1.25)1.30–1.93, Qm = 1.64 ± 0.19, elliptic, hyaline, smooth, inamyloid, thin-walled. Basidia (12)16–23 × 3–6 µm, clavate to cylindrical, 2- or 4-spored, hyaline, smooth, thin-walled. Cheilocystidia (16)17–28(29) × 3–6 µm, cylindrical to clavate with mamiform, often longer apical projections, less commonly weakly coralloid, hyaline, smooth, thin-walled. Pleurocystidia and caulocystidia not observed. Pileipellis a “*dryophila*-type” cutis, 8–14 µm wide, hyaline, smooth, thin-walled. Clamp connections present in all tissues.

##### Habit, habitat, and distribution.

Scattered to gregarious. Saprotrophic, with humicolous habitat, found in broad-leaved forests. So far, it is only known from Henan Province, China.

##### Other specimens examined.

China. Henan Province • Zhumadian City, Biyang County, Mingzhuang Village, 10 July 2021, Jia-Jun Hu, Bo Zhang, and Xiao Li, ZNU-F-002 (Collection No.: Hu 770) • Zhumadian City, Biyang County, Mingzhuang Village, 10 July 2022, Jia-Jun Hu, Bo Zhang, and Xiao Li, ZNU-F-003 (Collection No.: Hu 775).

##### Note.

This species is characterized by the basidiomata occurring in the summer, a dark reddish-brown pileus, a cylindrical to clavate stipe, an apex of cheilocystidia that is not diverticulate or lobate.

*Gymnopusbiyangensis* is similar to species in the *G.erythropus* complex due to the dark reddish-brown pileus. However, this species differs from *G.erythropus* by the light yellow and cylindrical to clavate stipe, smaller basidiospores, and a non-encrusted pileipellis. *Gymnopusbiyangensis* can be distinguished from *G.fagiphilus* by the smooth and light-yellow stipe and smaller basidiospores.

#### ﻿ Gymnopus sinodryophilus

Taxon classificationFungiAgaricalesOmphalotaceae

J.J. Hu, B. Zhang & Y. Li sp. nov.

C279EFAE-8B3E-550D-B62C-3D08B389C61E

Fungal Names: FN 572235

Figs 2B , 4

##### Etymology.

Refers to the species similar to *G.dryophilus*.

##### Diagnosis.

[English] This species is characterized by the yellowish-white to light-brown basidiomata, arisen from coniferous and broad-leaved mixed forest, coralloid but inflated pileipellis elements, and a non-coralloid apex of cheilocystidia.

**Figure 4.**
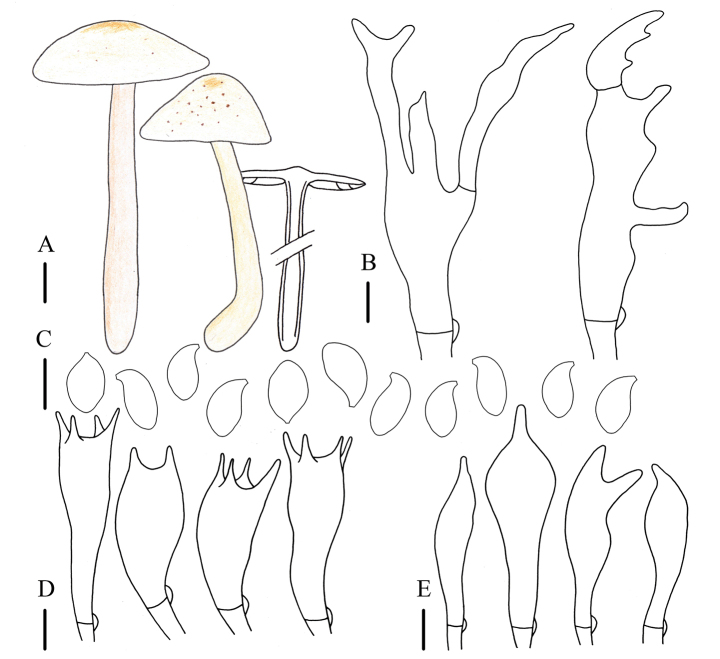
Morphological characteristics of *Gymnopussinodryophilus* (ZNU-F-003) **A** basidiomata **B** pileipellis elements **C** basidiospores **D** basidia **E** cheilocystidia. Scale bars: 1 cm (**A**); 5 µm (**B–E**).

##### Type.

China. Jilin Province • Changchun City, Jingyue District, Mt. Lianhua, 09 August 2021, Jia-Jun Hu and Bo Zhang, ZNU-F-005 (Collection No.: Hu 809), holotype.

##### Description.

Basidiomata small to medium. Pileus 1.0–4.4 cm in diameter, applanate-hemispheric to convex, light yellow to light brown, darker at center, brown, occasionally with brown spots, with an umbo sometimes, smooth, glabrous; margin entire, involute, yellowish white to light yellow. Context thin, fresh, white to light yellow, odorless. Stipe 1.3–8.8 cm long and 0.3–0.9 cm wide, central, clavate, light brown to brown, paler downwards, becoming light yellow to yellowish white, striped, smooth, glabrous, fistulose, fibrous. Lamellae adnexed, close to crowded, yellow to light brown, unequal. Occurrence in leaf litter in mixed forest.

Basidiospores (4.0)5.0–6.0(6.2) × 3.0–3.8 µm, Q = (1.33)1.39–2.00(2.07), Qm = 1.67 ± 0.18, elliptic, hyaline, smooth, inamyloid, thin-walled. Basidia (12)13–23 × 4–8 µm, clavate to cylindrical, 2- or 4-spored, hyaline, smooth, thin-walled. Cheilocystidia (11)13–27(31) × 3–8 µm, cylindrical to clavate, umbonate-mamiform with a short apical projection, occasionally forked, hyaline, smooth, thin-walled. Pleurocystidia and caulocystidia not observed. Pileipellis a “*dryophila*-type” cutis, (5)7–10(12) µm wide, hyaline, smooth, thin-walled. Clamp connections present in all tissues.

##### Habit, habitat, and distribution.

Scattered to gregarious. Saprotrophic, with humicolous habitat, found in mixed forests. So far, only known from Jilin Province, China.

##### Other specimens examined.

China. Jilin Province • Yanbian Korean Autonomous Prefecture, Antu County, Erdaobaihe Town, 26 June 2021, Jia-Jun Hu and Bo Zhang, ZNU-F-006 (Collection No.: Hu 743) • Changchun City, Jingyue District, Jingyuetan National Forest Park, 08 August 2021, Jia-Jun Hu, Bo Zhang, ZNU-F-004 (Collection No.: Hu 807) • Changchun City, Jingyue District, Jingyuetan National Forest Park, 08 August 2021, Jia-Jun Hu, Bo Zhang, ZNU-F-007 (Collection No.: Hu 811) • Changchun City, Jingyue District, Jingyuetan National Forest Park, 08 August 2021, Zheng-Hao Zhang, Jia-Jun Hu, Bo Zhang, ZNU-F-008 (Collection No.: Hu 892).

##### Note.

This species is characterized by the yellowish-white to light-brown basidiomata, which arise in summer; coralloid but inflated pileipellis elements; and a non-diverticulate apex of cheilocystidia.

This species is extremely similar to *G.dryophilus* due to the analogous morphology. However, this species differs from *G.dryophilus* by appearance in summer, smaller basidiospores, and the apex of cheilocystidia not being diverticulate.

### ﻿Key to the reported species of Gymnopussubsect.Levipedes in China

**Table:** 

1	Basidiomata with red stipe	**2**
–	Basidiomata with yellow stipe	**10**
2	Stipe covered with dense hairs at the base	**3**
–	Stipe smooth, or covered with sparse hairs at the base	***G.erythropus***
3	Basidia sterigmata extremely long	**4**
–	Basidia sterigmata short	**6**
4	Stipe smooth in upper part	**5**
–	Stipe covered with brown pruina on the upper part	***G.longus***
5	Pileus pale color, stipe color uneven	***G.longisterigmaticus***
–	Pileus dark color, stipe color uniform	***G.macrosporus***
6	Growing on the deciduous layer or rotten branches	**7**
–	Grows at the base of *Tilia* sp.	***G.tiliicola***
7	Pileus pale color, near white	***G.tomentosus***
–	Pileus deep color	**8**
8	Stipe covered with longitudinal striate	***G.striatus***
–	Stipe without longitudinal striate	**9**
9	Pileipellis a cuits, typically “*dryophila* type”	***G.changbaiensis***
–	Pileipellis layered, hyphae inflated to spherical to prolate	***G.globulosus***
10	Apex of cheilocystidia diverticulate	**11**
–	Apex of cheilocystidia not diverticulate	**19**
11	Basidiomata marasmioid	***G.striatipileatus***
–	Basidiomata collybioid or tricholomatoid	**12**
12	Cheilocystidia absent	***G.longistipes***
–	Cheilocystidia present	**13**
13	Caulocystidia present	***G.inexpectatus***
–	Caulocystidia absent	**14**
14	Pileus green	***G.viridiscus***
–	Pileus not green	**15**
15	Stipe cylindrical or clavate	**16**
–	Stipe enlarged at base	**18**
16	Stipe light red	***G.aquosus***
–	Stipe light yellow to yellow	**17**
17	Stipe covered with tomentose	***G.brunneodiscus***
–	Stipe smooth	***G.dryophilus***
18	Stipe pale yellow, basidiospores smaller than 6 µm	***G.dryophiloides***
–	Stipe light yellow to reddish brown, basidiospores larger than 6 µm	***G.ocior***
19	Basidiomata and lamellae light yellow	***G.sinodryophilus***
–	Basidiomata reddish brown, lamellae light reddish brown	***G.biyangensis***

## ﻿Discussion

In this study, two new species within the *G.dryophilus* complex are proposed. *Gymnopusbiyangensis* is characterized by summer-fruiting basidiomata found in broad-leaved forests, a dark reddish-brown pileus, a cylindrical to clavate stipe, and clavate to cylindrical cheilocystidia with a long apical projection. In contrast, *Gymnopussinodryophilus* is characterized by yellowish-white to light-brown basidiomata that arise from coniferous and broad-leaved mixed forests, coralloid but inflated pileipellis elements, and cheilocystidia with a short apical projection.

The sect. Levipedes is divided into two subsections: subsect. Alkalivirentes Antonín & Noordel. and subsect. Levipedes Antonín & Noordel., based on whether the mycelium turns green in potassium hydroxide (KOH) and ammonium hydroxide (NH_4_OH) (Halling 1981; Antonín and Noordeloos 1997).

Species from the *G.erythropus*, *G.fagiphilus* (Velen.) Antonín, Halling & Noordel., and *G.dryophilus* complexes primarily comprise subsect./ Levipedes. Our previous work demonstrated that the *G.dryophilus* complex species clearly differs from the *G.erythropus* complex species, particularly in terms of seasonal occurrence and the shape of cheilocystidia (Hu et al. 2022b, Hu 2023). The species of subsect.Levipedes are characterized by smooth, polished, or pubescent stipes; pileipellis is typically an entangled trichoderm (never radially oriented), composed of inflated, often lobed elements or coralloid, “*dryophila*-type” structures; trama and its elements are non-dextrinoid (Halling 1996; Antonín and Noordeloos 2010; Oliveira et al. 2019). However, in our study, *G.sinodryophilus* exhibited branched but not broadened pileipellis elements, which contrasts with the established boundaries. Similar deviations have been observed in *Gymnopusglobulosus* J.J. Hu, Y.L. Tuo, B. Zhang & Yu Li, *Gymnopusearleae* Murrill, and *Gymnopuskauffmanii* (Halling) Halling, etc. These findings suggest that the current limits and taxonomic framework of subsect.Levipedes require further examination.

Although the taxonomic history of *Gymnopus* (formerly *Collybia*) spans over two centuries, research on this genus remains considerably behind other groups, such as Amanitaceae (Cui et al. 2018), boletes (Wu et al. 2016), and Ganodermataceae (Sun et al. 2022). Although some species are edible or possess potential applications, such as in wastewater treatment (Sun et al. 2021a) and biological control (Wu 2016), they have not gained the same recognition as culinary fungi, for example, *Morchella* Dill. ex Pers. or *Tuber* P. Micheli ex F.H. Wigg. This lack of prominence may be a key reason why this genus has been overlooked in research. To truly understand this group, greater attention must be devoted to its study.

## Supplementary Material

XML Treatment for Gymnopus biyangensis

XML Treatment for Gymnopus sinodryophilus

## ﻿Additional information

### Author ORCIDs

JiaJun Hu https://orcid.org/0000-0002-7562-7612

Yong-Lan Tuo https://orcid.org/0000-0001-6019-1038

Zheng-Xiang Qi https://orcid.org/0000-0002-0037-9407

Yu Li https://orcid.org/0000-0003-4719-7210

Bo Zhang https://orcid.org/0000-0001-9508-8188

### Data availability

All of the data that support the findings of this study are available in the main text.
